# Genetic instability and anti-HPV immune response as drivers of infertility associated with HPV infection

**DOI:** 10.1186/s13027-021-00368-1

**Published:** 2021-05-10

**Authors:** Maria Isaguliants, Stepan Krasnyak, Olga Smirnova, Vincenza Colonna, Oleg Apolikhin, Franco M. Buonaguro

**Affiliations:** 1 N.F. Gamaleya National Research Center for Epidemiology and Microbiology, Moscow, Russia; 2 Chumakov Federal Scientific Center for Research and Development of Immune-and-Biological Products of Russian Academy of Sciences, Moscow, Russia; 3 Riga Stradiņs University, Riga, Latvia; 4 Department of Microbiology, Tumor and Cell Biology, Karolinska Institutet, Stockholm, Sweden; 5 Research Institute of Urology and Interventional Radiology named after N.A. Lopatkin, Moscow, Russia; 6 Engelhardt Institute of Molecular Biology, Russian Academy of Sciences, Moscow, Russia; 7 Center for Precision Genome Editing and Genetic Technologies for Biomedecine, Engelhardt Institute of Molecular Biology, Russian Academy of Sciences, Moscow, Russia; 8 Institute of Genetics and Biophysics “Adriano Buzzati-Traverso”, National Research Council, Naples, Italy; 9 Istituto Nazionale Tumori - IRCCS “Fondazione Pascale”, Naples, Italy

**Keywords:** Reproductive health, Infertility, Spontaneous abortion, Human papilloma viruses of high oncogenic risk type, Sperm cells, Oocyte, Gastrulation, Genomic instability, DNA damage, Viral integration

## Abstract

Human papillomavirus (HPV) is a sexually transmitted infection common among men and women of reproductive age worldwide. HPV viruses are associated with epithelial lesions and cancers. HPV infections have been shown to be significantly associated with many adverse effects in reproductive function. Infection with HPVs, specifically of high-oncogenic risk types (HR HPVs), affects different stages of human reproduction, resulting in a series of adverse outcomes: 1) reduction of male fertility (male infertility), characterized by qualitative and quantitative semen alterations; 2) impairment of couple fertility with increase of blastocyst apoptosis and reduction of endometrial implantation of trophoblastic cells; 3) defects of embryos and fetal development, with increase of spontaneous abortion and spontaneous preterm birth. The actual molecular mechanism(s) by which HPV infection is involved remain unclear. HPV-associated infertility as Janus, has two faces: one reflecting anti-HPV immunity, and the other, direct pathogenic effects of HPVs, specifically, of HR HPVs on the infected/HPV-replicating cells. Adverse effects observed for HR HPVs differ depending on the genotype of infecting virus, reflecting differential response of the host immune system as well as functional differences between HPVs and their individual proteins/antigens, including their ability to induce genetic instability/DNA damage. Review summarizes HPV involvement in all reproductive stages, evaluate the adverse role(s) played by HPVs, and identifies mechanisms of viral pathogenicity, common as well as specific for each stage of the reproduction process.

## Key points

- HPVs infect male and female genital tract, the infection is associated with various pathologies including the development of cancer. Replication of HPVs in dividing cells of the female and male genital tract can cause disruption of the cell cycle, cell proliferation, eventually immortalization, and may result in the malignant transformation of the infected cells.
- HPV infection is grossly involved in human infertility. The effect is site/tissue specific.
- The effects of HPVs on the cells of human reproduction system are differential (i.e. depend on HPV type). They involve mainly high carcinogenic risk HPVs (HR HPVs), such as HPV16 and HPV18.
- Male infertility associates with HPV infection of the semen. Specifically, HPV infection affects the quality of sperm cells, reducing their fertilization potential. HPV-infected sperm cells can transfer the virus to placenta and to the oocyte.
- Female infertility associates with HPV infection of the placental cells. HPV causes their miss-function, including compromised attachment of the trophoblasts. Placental cells can transfer HPV to the embryo.
- Transfer of HPV from the sperm cells or placenta into the embryonal cells causes damage and death of the infected oocyte/zygote/blastula/blastocysts, affects growth of the surviving embryonal cells, and altogether compromises early development of the embryo with consequent early pregnancy loss and early failure of assisted reproduction treatment.
- The direct pathogenic effects of HR HPVs are associated with the damage of host cell DNA by the reactive oxygen and/or nitrogen species generated in the course of the infection.
- DNA damage includes the induction of genomic instability in the form of polyploidy, chromosomal loss of heterozygosity, microsatellite instability, integration of HPV DNA full-length or in fragments into the genome of the infected gametes and embryonal cells, not (fully) repaired by the DNA damage reparation machinery compromised by HR HPVs. DNA damage reduces viability of the gametes, may cause their apoptosis, interfering with fertilization and early embryogenesis.
- Both male and female infertility may be caused by anti-HPV immune response built in the course of natural infection, mediating clearance of HPV-infected spermatozoa, oocytes, blastula and blastocysts up-to the rejection of HPV-infected embryo (maternal graft-versus-host disease against HPV infected fetus).
- HPV vaccination can prevent this type of infertility, and even reverse it for those already HPV infected.

## Introduction

**Fig. 1:**
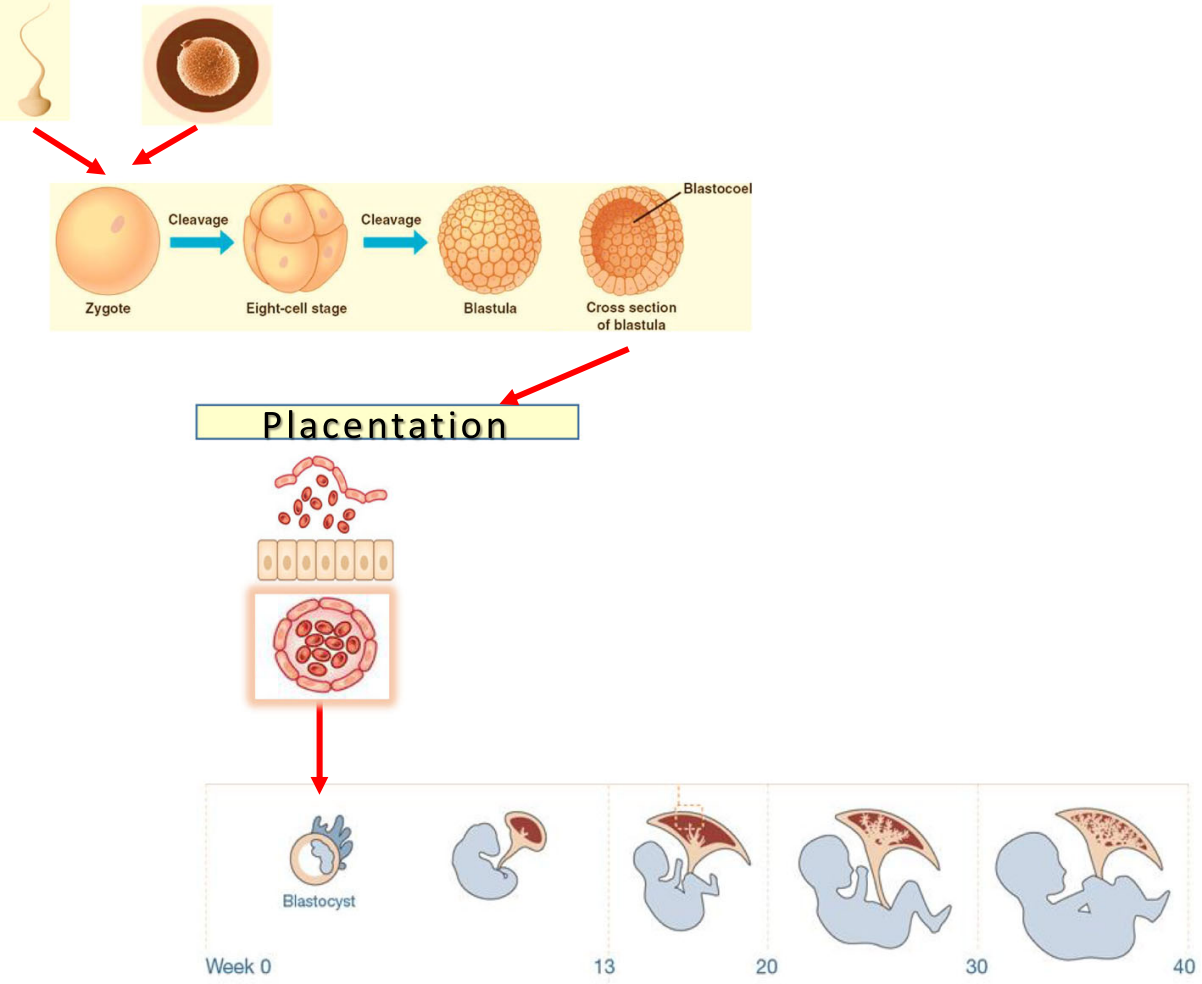
Schematic representation of the stages of fertilization and embryonal development. Infertility may originate from defects in production and/or functionality of gametes, fertilization, early development of the embryo, its placentation, as well as the late stages of embryonal development. Figure elements are adapted from [1, 2] and Human placental project at https://www.nichd.nih.gov/research/supported/HPP/form

Achievement of pregnancy implies successful completion of a long chain of successive events starting at gamete cells, spermatozoid and oocyte, continued to the zygote, blastula, and blastocyst, which after implantation into the uterine wall develops into the early multicellular embryo (gastrula) (Fig. 1). Germ tissue layers of the embryo, ectoderm, endoderm, and mesoderm, develop into the internal organs acquiring the distinct form of a fetus. During the first trimester of pregnancy, the outer layer of the embryo begins to merge with the endometrium and the placenta is formed. Placenta fulfills the nutrient and waste requirements and controls passive immunity of the embryo and fetus by passaging the maternal immunoglobulins [1].

Process fails if sperm cells cannot fertilize the oocyte, oocyte does not develop into embryo, embryo cannot implant, or fetus fails to develop, resulting in infertility (Fig. 1). Today, about 15% of all couples are infertile and seek medical treatment for fertility. According to the World Health Organization (WHO), the definition of infertility is ‘the inability of a sexually active, non-contracepting couple to achieve spontaneous or clinical pregnancy after 12 months of regular and unprotected sexual intercourse” [3]. Fertilization failure could be due to the paternal and maternal factors, and implantation and fetus development failure, to the maternal factors and/or embryonic causes. Achieving pregnancy requires treatment with assisted reproductive technologies. An adequate diagnosis of the infertility is of major importance to evaluate if it could be overcome and if the abnormality can be transmitted to the offspring.

A systematic analysis of 277 demographic and reproductive health surveys in 190 countries demonstrated that the total rate of female-associated infertility exceeds 12%, constituting 1.9% for primary, and 10,5% for secondary infertility. The levels are basically unchanged for the last 20 years [4]. Female infertility, in the context of assisted reproductive technology, is split into several etiologies including dysfunctions of reproductive apparatus: tubal factor, ovulatory dysfunction, diminished ovarian reserve, endometriosis and uterine factor [5]. The other common causes are advanced age, sexually transmitted diseases and immunological factors [6]. Male-infertility-associated factor(s) together with the abnormal semen parameters are identified in up-to 50% of infertile couples [7]. Known causes of male infertility are: congenital or acquired urogenital abnormalities, malignancies, urogenital tract infections, increased scrotal temperatures, endocrine disturbances, and immunological factors. The most common abnormalities in observed in a routine semen analysis are: absence of spermatozoa (azoospermia); very low sperm count in the ejaculate (oligozoospermia); abnormal sperm morphology (teratozoospermia) and/or abnormal sperm motility (asthenozoospermia).

Between 15 and 30% of infertile men and ≈ 10% of infertile women display genetic abnormalities, including chromosome aberrations, single- or multiple-gene mutations, and polymorphisms, as well as mitochondrial and epigenetic disturbances, which result in so called idiothypic infertility with human genetic aberrations as the main suspected cause [8]. Number one among the genetic infertility causes is polycystic ovary syndrome (PCOS). PCOS is a complex and heterogeneous endocrine condition that affects 5–10% of women [9]. Another genetically predetermined reproductive disease is premature ovarian failure (POF). POF is defined as the onset of menopause in women under the age of 40 years, associated with mutations in nine host genes [9]. Non-syndromic male infertility was associated with the Genome-Wide Association (GWAS), Online Mendelian Inheritance in Man (OMIM) and differentially expressed genes (DEG) genes, in total, 168 GWAS/OMIM/DEG genes with specific mutations identified in 31 distinct genes. These genes play an important role in metabolism, genetic information processing (DNA replication, repair and transcription), RNA degradation and translation, protein folding, sorting and degradation, processing of environmental information, cellular processes and human diseases such as cancer, drug resistance, substance dependence, and endocrine, metabolic, cardiovascular, immune, degenerative and infectious, including viral, diseases [10–13]. Despite interesting data provided by GWAS and continuous increase in the number of infertility associated genes/patterns of genes and single mutations, extensive longitudinal efforts to identify the recurrent genetic factors with potential clinical application were not successful [14], the actual causes of the idiopathic infertility in many females and males remain largely unknown. All in all, the etiology of infertility remains unidentified in up to 13% of cases in women and 30 to 40% of cases in men [5].

## HPV infection and human reproduction

Human papillomavirus (HPV) is a sexually transmitted pathogen commonly detected in men and women of reproductive age worldwide. Chronic infection with HPVs, specifically of high carcinogenic risk types (HR HPVs), are associated with epithelial lesions and cancers, as well as many adverse effects in the reproductive function. The majority of genital HPV infections are likely to be caused by the genital-to-genital sexual transmission [15]. There are other ways of horizontal transmission, such as breastfeeding [16], and hand-genital contact considered to be rare [15]. HPV is also transmitted vertically via transplacental and intranatal routes [17].

In the acute phase, HPV infects basal cells of the stratified epithelium through micro wounds in the epithelial barrier that remove the full thickness epithelium, but retain the basement membrane. Virus, via its L1 protein, binds first to the basement membrane and then to the cellular receptor on the migrating wound keratinocyte [18]. Putative HPV receptors on the basement membrane are alpha-integrins, laminins, and annexin A2. HPV bound to the receptors enters cells by endocytosis. Thereafter, HPV persists in the infected cell without killing it with episomal copies of its genome attached to host chromatin and replicated together with host DNA [19]. Newly assembled viral particles are released with desquamating cells. Virus is transmissible as soon as it infects basal epithelial cells. Replication of HPVs in dividing epithelial cells causes disruption of the cell cycle, cell proliferation, eventually immortalization and may result in the malignant transformation of the infected cell and development of cancer [20]. Direct pathogenic effects of chronic HPV infection are: the induction of genomic instability in the form of polyploidy, chromosomal loss of heterozygosity, microsatellite instability, integration of HPV DNA full-length or in fragments into the genome of the infected cell with acquisition of mutations causing or predisposing to malignant transformation [19, 21, 22].

It has long since been observed that HPV infection of partners (couples) leads to an increased risk of inability to conceive and pregnancy loss. A study of 106 subjects demonstrated that HPV positive women had a decreased pregnancy rate (4 of 17, 23.5%) as compared with HPV-negative women (51 of 89, 57.0%, *P* < .02) [23]. Furthermore, of 590 women who had undergone intrauterine insemination (IUI), those with an HPV infection had six times less pregnancies compared to those who tested negative [24]. Interestingly, Depuydt CE et al. also looked for possible effects of infection with Trichomonas vaginalis and Chlamydia trachomatis on pregnancy outcome; the former was not detected, and the latter did not have any impact on the pregnancy rates [24]*.* The follow-up of pregnancies showed a higher miscarriage rate in HPV infected versus uninfected couples (62.5% vs. 16.7%) [25]. Increased incidence of pregnancy loss has also been demonstrated in in-vitro fertilization (IVF) with HPV-infected semen of male partners, when HPV is localized in the sperm cells [26]. Analysis for HPV DNA in first trimester spontaneous and electively aborted products of conception showed three-times higher prevalence of detection of HPV16 E6/E7 DNA (amplification of E6/E7 junction region) in spontaneous versus electively aborted fetal materials (15/25 or 60% versus 3/15 or 20%, respectively) [27]. These data point at the gross involvement of HPV infection in human infertility depending on the paternal, maternal factors and embryonal causes. The purpose of this review is to analyze vast data accumulated in the field in attempt to dissect the molecular mechanisms of HPV-associated infertility.

## Epidemiological association of HPV and reproductive stages*:* HPV-related male infertility

### HPV infection of male genital tract and semen

The main mode of entry of HPV into the male genital tract (MGT) is through the penile mucosa. In MGT, HPV virions localize to the perianal region and external genitalia, including the penis foreskin, scrotum and glans penis, urethra, ductus deferens, epididymis, and testis [28]. Male circumcision decreases the prevalence of HPV in men, including high-risk, and has been associated with reduced acquisition of the virus as well as with increased viral clearance. These data suggest that the foreskin constitutes a favorable environment for HPV infection [29]. The rate of HPV infection of other areas of MGT is lower.

HPV is often detected in the semen/seminal fluid. Pooled prevalence of HPV DNA in semen reaches 16%, varying from 0 to 100% [30]. Meta-analysis of 31 case-control studies of infertility done in the period 1990–2016 based on the PRISMA guidelines demonstrated negative effect of HPV on the fertility in case of virus localization in the semen [30]. Meta-analysis estimated HPV prevalence among general male population and males attending fertility clinics (in total 5194) in 16 countries in Europe, North and Latin America, Asia, Oceania, and Africa. HPV DNA in the semen in general population and in the attendees of the fertility clinics was found in 11.4 and 20.4%; DNA of HR HPVs in 10.0 and 15.5%; and HPV16 DNA in 4.8 and 6.0%, respectively, demonstrating a significantly increased risk of infertility for males positive for HPV DNA in semen [30]*.* HPV16 was the most common HR HPV found in the semen, second most common was HPV56 (infrequently seen in HPV-related cancers) [30]. HPV DNA could be found in every fraction of the semen: seminal plasma, exfoliated, immune cells and in varying proportion of the semen cells [31].

It is widely accepted that seminal HPV originates from the genital skin and mucosa [32, 33]. The latter is supported by significant associations between HPV types found in the semen and on the genital skin [33, 34]. The other source is the urogenital tract reservoirs, specifically the testis. Several studies examining the prevalence of HPVs in the testis and epididymis, found it to vary from 0 to > 30% [34–38]. HPV infection of the testis could affect fertility in two ways. Firstly, it may result in the changes of the testicular function, representing a serious risk for the fertility and general health of the individual. Indeed, higher prevalence rates of HPV in testis and epididymis were associated with pathologies of MGT such as nontuberculous epididymitis [36] and azospermia [39]. Secondly, it may lead to HPV infection of sperm cells. Indeed, the seminiferous tubules of the testis support the development of germ cells into the haploid spermatozoa. This process starts with the mitotic division of the spermatogonial stem cells (SSC) located close to the basement membrane of the tubules. HPV can then be transferred from the testis/basement membrane of the tubules to the stem cells resulting in the generation of HPV-infected stem cell progeny. HPV infection of SSC implies inheritance by the sperm cells of HPV DNA, full length or truncated, inserted into the host genome, as well as the inheritance of other genetic aberrations caused by HPV infection of SSCs, with genetic defects eventually transferred to the fertilized egg, affecting development of the embryo. Transfer of HPV from SSC precursors implies infection of a high percent (or all?) sperm cells with HPV resident in SSC. Experimentally, Foresta C et al. found HPV virions in a proportion of semen cells [40]. Images of in situ hybridization of spermatozoa detecting HPV virions reveal that this proportion could be quite high [41], but it does not reach 100%, as one can expect if all spermatozoa develop from HPV-infected precursors, in which episomal HPV DNA is replicated together with the chromosomes of the host, supporting the infection of sperm cells though the genital skin and mucosa. The actual role of spermatogonial stem cell infection with HR HPVs in generation and functionality of HPV-infected sperm cells has yet to be elucidated.

### Effect of HPV on male fertility depends on the type of infected semen cells

**Fig. 2:**
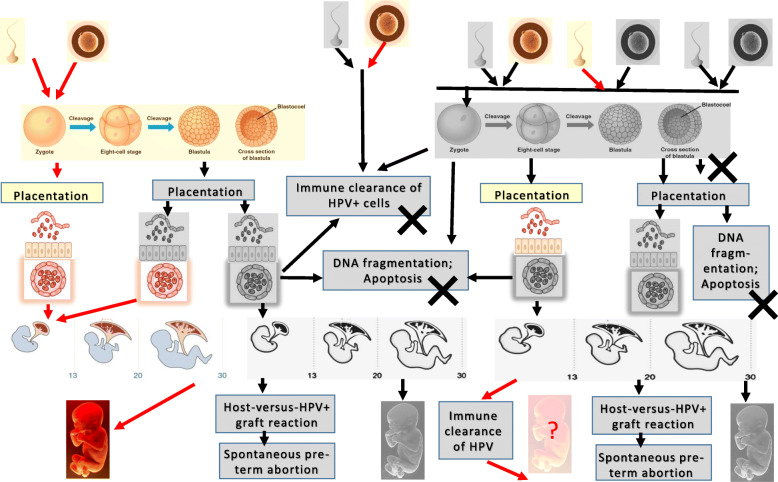
Stages of the fertilization and embryonal development compromised by HPV infection. Cells, tissues and embryo infected with HPVs at different stages of development are colored in grey tones. Black cross designates preterm termination of the embryonal development. Spontaneous clearance of HPVs from the epithelial tissues is well documented; no data exists on possibility of HPV clearance from the infected embryonal cells and/or fetus, this possibility is not excluded and is designated on the scheme with a question mark. Elements of the figure are adapted from [1, 2], Human placental project at https://www.nichd.nih.gov/research/supported/HPP/form and image of five month human fetus corresponding to the successful passage of 20 weeks of pregnancy (no spontaneous/preterm abortion), from https://www.pinterest.com/pin/441352832202220770/

HPV DNA is detected in every fraction of the semen: spermatozoa, somatic cells and seminal plasma. Virus could be localized just in the sperm cells, or just in the exfoliated cells, or in both [26]. Additionally, different fractions may contain multiple HPV genotypes in varying quantities, with several HPV genotypes in one and the same fraction [31]. Infertility depends on what fraction/which cells are HPV infected. Study done on 226 infertile couples demonstrated a success of intrauterine insemination (IUI) and intra-cytoplasmic sperm injection (ICSI) treatments in 38% of couples if semen was HPV free, and only in 14% if semen was HPV infected, i.e. a significant reduction of pregnancy rates for couples where male partner had HPV DNA positive semen [25]. Similarly, HPV infection was found to grossly affect the outcome of insemination with donor sperm. Testing of sperm banks demonstrated that 3.9% (20/514) of tested donor sperm is HPV positive (3.6% bank A, 3.1% bank B and 16.7% bank C); HPV virion load per spermatozoon across different sperm banks was similar (from 0,01 to 1,07 HPV virions per spermatozoon). While pregnancy rate with HPV negative donor sperm reached 14.6%, insemination with HPV positive donor sperm did not result in clinical pregnancies [42]*.* Thus, HPV infection of the male partners, namely of their sperm cells/spermatozoa, significantly reduces pregnancy rate in both spontaneous pregnancies and insemination, i.e. affects the fertility (Fig. 2).

### The effects of HPV infection on the properties of sperm cells

HPV infection affects male fertility in different ways. Already early studies demonstrated that HPV16 and 18 are able to transcribe in the infected sperm cells [43], which are then subjected to the adverse effects of HPV proteins, including significant impairment of sperm quality. Lai et al. was the first to report the malfunctioning of the sperm infected with either of 12 HR HPV types, observed as lower curvilinear velocity, straight-line velocity and mean amplitude of lateral head displacement [44]. These early findings were corroborated by the studies of Foresta C et al. who found that infected sperm samples are often characterized by the impairment of sperm parameters, such as cell concentration, morphology, and pH resulting in the reduced progressive sperm motility [45, 46].

The data on sperm motility alterations due to HPV infection is, however, contradictory. A study of semen samples found 24 of 308 semen samples (7.8%) to be positive for either of HPV6/11/16/18/31/33/35, but HPV infection did not seem to affect semen quality, their role in male infertility could not be demonstrated [41]. Neither could it be reproduced in a study of semen in 340 Slovenian men from infertile couples scanned for 37 HPV types including 12 HR HPVs [47]. The effect of HPV infection on sperm motility could not be conclusively confirmed even after an extensive meta analysis of the data collected in 10 studies done in Italy, China, and Iran which collectively found a broad spectrum of HPV types in 616 out of 2645 samples [48]. There were indications that sperm progressive motility was significantly reduced in the HPV-infected semen samples compared to the non-infected groups [SMD:-0.88, 95% CI:-1.17 ~ − 0.5], but the level of statistical heterogeneity was alarmingly high (I2 value: 86%) [48]. A series of alterations in sperm quality was detected after semen exposure to DNA of HPV6, 11, and HR HPVs 16, 18, and 33 [49]. However, washed sperm motility was actually not lower, but higher in the presence of DNA of all HPV DNA types except HPV 6/11. To conclude, there exist other (than motility) aspects of sperm quality, or rather, other properties of sperm cells acquired due to HPV infection that affect fertilization.

Other sperm cell alterations caused by HPV infection are reduced viability, decreased cell counts, decreased amount of cells with normal morphology and, importantly, DNA damage [50]. In support of the latter, exposure of sperm cells to HPV DNA (except HPV18) increased sperm linearity; furthermore, DNA of HPV16 and HPV31 were found to cause significant fragmentation of sperm DNA [49]. Altogether, these data demonstrate that HPV infection affects the quality of sperm cells in different ways, which cumulatively lead to their reduced capacity to fertilize the oocyte. These findings also indicate that the effects of HPVs on the quality of sperm are differential (i.e. depend on HPV type) and therefore cannot be revealed in a meta analysis which does not distinguish between HPV types. The exact nature of these differential HR HPV-type specific alterations, and molecular mechanism(s) by which HPVs (specifically HR HPVs) can cause them, remain to be elucidated.

### Final act: transfer of HPV from sperm cells into the oocyte

Pathological alterations can also occur at the final stage of fertilization, the fusion, due to the transfer of HPV from the infected sperm cell into the egg (Fig. 2). In spermatozoa, in situ hybridization (ISH) reveals clear HPV localization at the equatorial region of the sperm head [41]. The equatorial segment (the EqS) of mammalian sperm is the site for sperm-egg fusion initiation, and the organizing center for assembly of molecular complexes required for gamete interaction and fusion. Sperm-egg membrane fusion involves three tetraspanins, CD9 and CD81 and, as lately discovered, also CD151 forming a novel tetraspanin network [51]. CD151 is highly expressed in the basal layers of cervical mucosa, where epithelial cells come into direct contact with the basement membrane. In testicular cell subpopulations, CD151 gene and protein expression shows strong enrichment in spermatogonia and spermatids. In the sperm cell, CD151 is located into the inner acrosomal membrane overlying the nucleus. The testicular and epididymal localization pattern is specifically enriched in the EqS as the primary sperm head fusion site. Prior to gamete fusion, CD151 interacts with α6 integrin subunit, which forms a dimer with β4 as a part of cis-protein interactions within sperm [51].

Infectious entry of HPVs into cells occurs via the clathrin- and caveolin-independent endocytic pathway, which involves tetraspanin proteins and actin; tetraspanin web couples HPV contact site to the intracellular endocytic actin machinery and serves as a virus entry platform, co-internalized with the virus particle [52]. CD151 controls activities of the associated integrins [53]. Expression of the CD151-associated integrins (α3β1 and α6β1/4 integrin) is largely found in the basal keratinocytes [54]. These CD151-associated integrins α6β4 serve as the receptor for HPV16; HPV16 infection of keratinocytes critically relies on the formation of integrin-CD151 complexes, as was demonstrated by α6β1/4 short interfering RNA (siRNA) gene knockdown experiments [55]. Interaction of HPV16 with CD151 requires the intact C-terminal cytoplasmic region of the protein; overexpression of CD151 mutants unable to interact with integrins does not enhance HPV16 infection [56]*.* Thus, the tetraspanin network, specifically CD151, is tightly involved in both sperm-egg fusion and in the HPV infection of the target cells (keratinocytes).

During the process of infection of keratinocytes, HPV16 moves together with CD151 within the plane of the membrane to get co-internalized into the endosomes [56]. Viral particles bind CD151 on the cell surface, remain bound to CD151 during their lateral movement on the cell surface, but disappear after internalization; disappearance is seen only for CD151-bound HPV particles, while unbound virus remains on the cell surface [56]. Depletion of endogenous CD151 results in the reduction of HPV16 endocytosis, while not affecting binding of viral particles to cells. In addition, CD151 knockdown leads to a marked decrease in the number of HPV16 capsid-positive endosomes, thereby suggesting involvement of CD151 in both endocytosis and HPV16 disassembly [56]. Integrins α3β1 and α6 in complex with CD151 function in a post-binding step(s) of viral infection, possibly, as secondary HPV receptors [48]*.* Altogether, this indicates that interaction of HPV16 with CD151 and integrin α6 within the EqS facilitates an efficient transfer of HPV into the oocyte during fusion of gametes.

Another well described mechanism of HPV transfer from sperm cell into the oocyte involves interaction between the HPV capsid protein L1 and syndecan-1 [57]. Both sperm transfected with HPV E6/E7 genes and sperm exposed to HPV L1 capsid protein are capable to penetrate the oocyte and transfer the virus/viral genes into the oocytes [57].

HPV-infected sperm serves as a vehicle for HPV transfer. This is vividly supported by the observation of the placental HPV infection (with HPV16 and 62) occurring in previously uninfected women in early pregnancy [58]. Penetration of the HPV infected sperm cell into oocyte results in the intracellular delivery of HPV genome followed by active transcription of HPV genes in the fertilized egg [26, 57]. Thus, HPVs infect MGT, the infection is associated with various MGT pathologies (including cancer). Furthermore, HPV infection affects the quality of sperm cells, reducing their fertilization potential. The last but not the least, HPV-infected sperm cells can transfer the virus to placenta and to the egg, the consequences of this transfer will be dissected in the following chapters dealing with infertility in women.

## Epidemiological association of HPV and reproductive stages: HPV infection of women and infertility

### HPV-associated infertility in women depends on the site of HPV infection: focus on placenta

Published data point at a gross involvement of HPV infection in infertility in women [23, 24, 27]. Among HPV-positive women, 27.3% reported at least one previous pregnancy loss compared to 17.43% among HPV-negative women [59]. There are, however, studies with the opposite findings [59]. Case control study including 281 Mexican women with spontaneous abortion attending for curettage, and control pregnant women attended for delivery [59]. HPV molecular detection and typing of HPV16, 18, 58 and 6/11 was performed on cervical samples, with HPV16 and 58 most frequently detected in both groups, and multiple HPV types found in 31.4% of HPV-positive samples. Interestingly, however, HPV cervical infection was found to significantly associate with alcohol intake before the pregnancy and with multiple sexual partners, but not with the spontaneous abortions. In their turn, spontaneous abortions were associated with the previous losses of pregnancies and with women’s age older than 35 years, but not with positivity for HPV (or any of the TORCH agents detected as IgM against T. gondii, CMV, HSV) [59].

Systematic analysis of the data on HPV prevalence and pregnancy outcome in PubMed and Embase casted light on the actual correlates between women positivity for HPV and spontaneous abortions [60], specifically, its dependence on the nature of the tissues subjected to HPV DNA testing. The overall HPV prevalence in the normal full-term pregnancies constituted 5.7 to 17.5% for cervix, and 8.3% for placental tissue (also 5.7% for amniotic fluid, and 10.9% for umbilical cord blood). In the cases of spontaneous abortions and spontaneous preterm deliveries the figures were significantly higher, 24.5 and 47%, for the cervix; and 24.9 and 50% for placenta, respectively. Prevalence of HPV in placenta was specifically higher in cases of spontaneous abortions compared to the normal full-term pregnancies [60]. This meta analysis supported the data of an early observation of a more frequent detection of HPV DNA in the placentas from the spontaneous preterm deliveries compared to the placentas from controls (*P* = 0.03), whereas prevalence of HPV in placentas from cases of pre-eclampsia was not significantly different to the controls [61]. Thus, as in men, HPV-associated infertility in women depends on the site of HPV infection: HPV infection of the cervix appears to be irrelevant for the fertility, while HPV infection of placenta has an adverse effect on the pregnancy outcome.

### HPV infection of placenta, the role of extravillous trophoblasts and Hofbauer cells

Placenta, membranes, amniotic fluid, and fetus have been considered sterile for most of the last century. Changes in technology and greater appreciation of the human microbiome have questioned this dogma [62]. Syncytiotrophoblasts form a continuous barrier between the maternal and fetal circulation, they are relatively resistant to viral infection. However, undifferentiated, extravillous trophoblast cells are susceptible to infection with adenovirus [63] *.* This positions them as “the Trojan horses” within the placenta serving as the entry gate for placental invasion with the adverse reproductive outcomes. Already in 2008, Gomez LM et al. has shown that HPV infection of extravillous trophoblast induces cell death which may affect placental invasion into the uterine wall [61]. Lately, in situ hybridization identified HPV DNA in the cells of the placental villi mesenchyme in encasing endometrium, but predominantly in the trophoblast cells [64]. During the process of placentation, invasive extravillous trophoblasts migrate into the maternal uterus and modify its vessels; failures in this modification result in the pregnancy complications including recurrent abortion [65], HPV infection could be the cause of such failure, i.e. malfunction of the HPV-infected invasive extravillous trophoblasts.

HPV DNA was also detected in the Hofbauer cells [64]. Hofbauer cells (HBC) play an important role in the placental development including vasculogenesis and angiogenesis in the first trimester. Since HBC are macrophages, it has been assumed that these cells protect the placenta and fetus from infection*.* However, there is no experimental evidence that they are capable of killing microbes within the placenta [66]. During placental inflammation HBC may produce pro-inflammatory cytokines or mediators that damage the villous cell barrier, and induce fibrotic responses and chronic inflammation within the villi [66]. HBC can serve as viral reservoirs within the placenta; they were shown to support replication of Zika virus [67, 68] and were proposed to participate in vertical transmission of the virus from placenta to fetus [39, 69]. Further studies questioned the infectivity of Zika virions produced in HBCs, but other microorganisms harbored by HBS were referred to as viable [66]. As an example, respiratory syncytial virus progeny remains trapped within infected HBC for up to 30 days, with no release into surrounding media. HBC carrying live virions will then pass the infection onto overlaid naïve epithelial cells, suggesting contact-dependent trans-infection [70]. This may potentially happen if HBC are infected with HPV with resulting chronic inflammation affecting placental villous growth and tissue remodeling*.* The latter study confirmed that infected HBC can serve as a source of infection to the fetus.

### HPV infection and the process of embryogenesis

HPV contained in the infected sperm cells can infect both placenta and the oocyte (during fertilization). Presence of the virus in the fertilized egg would inevitably affect its development. Indeed, transfection of blastocysts with the E6-E7 region of HPV16 (but not HPV18, or 31, or 33) caused fragmentation of DNA, and subsequent trophoblastic death (see systematic review by Gizzo S et all [71]). DNA fragmentation causes apoptosis. Apoptosis rate in trophoblastic cells transfected with HPV16 was found to be 3-fold higher on day 3, and 5.8-fold higher on day 12 post transfection, as compared to the negative controls [61]. Furthermore, the surviving transfected blastocysts demonstrated a progressive loss of the invasion ability [61]. HPV16-infected trophoblasts also demonstrated an early (two-cell embryo stage of development) reduction of the growth rate [72]. Reduced growth rate and invasiveness, and eventually apoptosis, would lead to the spontaneous abortion, even before the pregnancy is documented. In this way, HPV infection of the oocyte acquired from the female genital tract, or from the infected sperm cell, or HPV infection of the blastocyte acquired from the placenta, gets directly involved in the female infertility (Fig. 2).

To summarize, HPV infection of the placental cells causes their miss-function, including compromised attachment of the trophoblasts, as well as transfer of HPV to the embryo/fetus, damage and possibly death of the HPV-infected oocyte/zygote/blastula/blastocysts resulting in the early pregnancy loss and early failure of IVF (Fig. 2).

## Common molecular mechanisms of HPV-associated infertility in men and women

### Infertility and cancer, two sides of one medal?

A study of over 64,000 women of childbearing age in the USA has found that infertility is associated with a higher risk of developing cancer compared to a group of over three million women without fertility problems, their risks to develop cancer significantly differed - 2% versus 1,7%, respectively. Specifically, this involved uterine, ovarian, lung, thyroid, liver and gallbladder cancer and leukemia [73]. The causes might be the infertility treatment, or infertility itself, or/rather, the common causes of infertility and cancer.

The data existing today indicate that infertility is non-random in the population and suggest that different infertility etiologies are not isolated and are not an exclusive disease of the reproductive system/hypothalamic–pituitary–gonadal axis, and not isolated consequences of certain specific mutations, but are genetically and clinically linked with other diseases into distinguishable meta-diseases [13]. Specifically, the infertility condition in women appears to be tightly linked to the endometrial [74] and ovarian cancer [75–77], specifically, its serous borderline, serous invasive, endometrioid and clear cell histological subtypes [78]. In men as well, infertility is significantly associated with multiple forms of cancer of urinary and reproductive systems [13]. This shifts the focus of the studies on the possible common molecular factors or rather, inductors, of infertility and cancer.

HPVs of high oncogenic risk (HR HPVs) are the causative agents of virtually all cases of cervical cancer as well as a significant percentage of other anogenital and oropharyngeal cancers. In fact, current estimates indicate that HPV infection may be associated with as many as 93% of the anal, 40% of penile, 64% of vaginal, and 51% of vulvar cancers [79]. The high-risk types encode two viral oncogenes, E6 and E7, which work together to initiate cell transformation [20]. Progression from HPV infection to cell transformation and further to cancer proceeds through multiple steps involving activities and interactions of viral and cellular proteins. Mechanism(s) driving HPV-associated malignant transformation could be the same or similar to the ones causing idiopathic infertility. One of the common mechanisms driving infertility and cancer could be oxidative and nitrosative stress manifested by the production of reactive oxygen and nitrogen species (ROS, RNS) with subsequent DNA damage.

### Reactive oxygen and nitrogen species and functions of the reproductive system

ROS are essential for attaining functional competence of the cells of reproductive system playing an important role in their physiologically. Of the cohort of oocytes developing in the ovary, only one, the dominant oocyte, proceeds to meiosis I. This process is regulated by ROS and antioxidants. Further progression of meiosis II is promoted by antioxidants, demonstrating a complex relationship between ROS and antioxidants in the ovary. ROS produced by the pre-ovulatory follicle are considered important inducers of ovulation, with inhibition of ROS disturbing the process [80]. Besides, ROS play an important role as secondary messengers in many intracellular signaling cascades involving the female genital tract [81]. In MGT, ROS are required for the formation of disulfide bonds between cysteine residues in protamines for sperm nuclear chromatin condensation during spermiogenesis. Besides, H_2_O_2_ activates formation of the protective mitochondrial capsule in mature sperm. Furthermore, ROS regulate the capacitation processes, the priming process that spermatozoa undergo in the female genital tract, and further hyperactivation and acrosomal reaction. Addition of ROS-generating materials, such as xanthine, xanthine dehydrogenase/oxidase, glucose oxidase (β-D-glucose:oxygen 1-oxido-reductase), NADPH and H_2_O_2_, can stimulate sperm to undergo hyperactivation, whereas addition of the antioxidant enzymes, such as catalase or superoxide dismutase, inhibits the sperm capacitation process and reduces the fertilizing potential [82].

RNS are especially prominent in the male reproductive system originating from various cell types such as seminal ejaculate, accessory glands, epididymis, penis, testes, and ducts [83]. At physiologic levels, RNS are crucial for various functions within the male reproductive system. Capacitation of spermatozoa in the female genital tract involves NO-mediated tyrosine phosphorylation of two sperm proteins. Of known RNS inducers, testis-specific subclass of neuronal nitric oxide synthase (NOS), known as TnNOS, localized solely in the Leydig cells of the testis, is involved in steroidogenesis; inducible NOS (iNOS) is associated with maintenance of germ cell number in the seminiferous epithelium; endothelial NOS (eNOS) and iNOS structurally associate with occludin, actin, alpha-tubulin, vimentin, controlling tight junctions in the testis and blood-testis barrier [83].

Thus, both ROS and RNS control fertilization on the overall. At the same time, the excessive amounts of ROS and RNS are harmful to the reproductive system [65, 82, 83]. Among the adverse effects, the excess of ROS (and RNS) can inflict serious damage to DNA of the gametes, including point mutations, polymorphisms, deletions, chromosomal rearrangements, frame shifts and single-stranded or double-stranded breaks [84].

### HPV infection, oxidative stress and DNA damage in the infected epithelial cells

During the last two decades it has been clearly established that ROS and RNS produced in viral infection act as powerful promoters of cell transformation and cancer development [85]. We and others have extensively described the role of chronic viral infections and of individual viral antigens in the induction of oxidative stress [86–89]. Both oxidative and nitrosative stress may be caused by the continuous expression of certain viral proteins as well as inflammatory immune response to viral infection. This is particularly true for the infections caused by the blood-borne hepatitis viruses (B, C, and D), HIV-1, influenza A, Epstein-Barr virus, respiratory syncytial virus, and other viruses. Human papilloma viruses are actively involved in the induction of oxidative stress. Patients with HR HPV infection have increased serum levels of malondialdehyde (MDA), serum marker of peroxidation of unsaturated fatty acids, compared to uninfected controls (4.56 ± 1.64 nM and 1.64 ± 0.37 nM, respectively). For oxidative damage of DNA, the most prone to oxidation is guanosine, radical hydroxyl attack to the eighth position of the guanine moiety results in formation of 8-Hydroxyguanosine (8-OHDG). During DNA repair processes this compound is released and is urinated without metabolism [90]. The levels of 8-OHDG in urine of HPV-infected are significantly higher than in uninfected (14.61 ± 1.39 ng/ml compared to 9.66 ± 1.74 ng/ml, *P* < 0.001) [91].

Oxidative stress caused by HPV infection is due to the activity of several viral proteins, specifically those, belonging to HR HPV types. Early protein E2 of HPV16 and HPV18 moves between the nucleus and the cytoplasm; the occurrence of E2 on the mitochondrial membranes increases the production of mitochondrial ROS. This phenomenon is not observed for HPVs of low/no oncogenic risk [92, 93]. The major role in the induction of oxidative stress and production of ROS is played by HR HPV oncoproteins E6 and E7 involved in cell transformation. Expression of E6 and E7 is sufficient to induce ROS generation in head and neck cancer cells (isogenic human cell model) [94]. Expression of one of the isoforms of HPV16 oncoprotein E6 increases the levels of ROS in both HPV-positive and HPV-negative cells, resulting in DNA damage, reliably detected by several assays. The observed effects could in part be explained by the E6-induced decrease in the cellular antioxidant activity, as the expression of this E6 isoform led to a decreased expression of superoxide dismutase isoform 2 and glutathione peroxidase [95]. Due to their state of chronic oxidative stress, HPV-positive cells are more susceptible to DNA damage induced by other agents, such as ionizing radiation [94]. Furthermore, several studies demonstrated that modulation of oxidative stress by E6 and E7 oncoproteins of HR HPV types leads to the accumulation of mutations predisposing to malignant transformation of the infected cells (see Silva GAF et al. for the review [96]).

E6/E7-induced oxidative stress is mediated by nicotinamide adenine dinucleotide phosphate oxidases (NOXs) [94]. NOX2 silencing significantly reduces generation of ROS, DNA damage and chromosomal aberrations in HPV-positive cells. Interestingly, NOX-related mechanism for genomic instability distinguishes HPV-positive from HPV-negative tumors, as NOX-induced oxidative stress is observed in HPV-positive but not in the HPV-negative cancer cells [80]. Besides, HPVs interfere with different elements of the antioxidant and DNA damage response (DDR) systems [97] up-to hijacking of the DNA damage response for viral replication [98]. Altogether, HR HPVs and their proteins are strongly involved in the induction and maintenance of the oxidative stress in the infected cells.

Interestingly, DNA damage inflicted by the activity of HR HPV proteins affects not only genomes of the semen cells and oocytes, but also of HPV itself. Capra et al suspected recombination between HPVs infecting one and the same cell of the semen [31]. However, careful analysis of the data indicates that they might have registered fragments of HPV genomes integrated into the genome of the host. Indeed, study by Leonard SM et al revealed disrupted high-risk HPV DNA in morphologically normal cervices of older women [99]. Ventana ISH detected HR-HPV in 42% of the study population, which also tested positive for HPV16 in the PCR based assays, with majority of study subjects having a history of preceding cytological abnormality. However, analysis of the subsets of this population revealed HR-HPV to be transcriptionally inactive (as there was no evidence of a productive or transforming infection); also E2 gene was always disrupted [99] indicating that ISH was detecting short integrated but not the full-length exosomal viral sequences.

### HPV infection, oxidative stress and DNA damage in human reproductive system

Oxidative stress in HPV infection would lead to the oxidation of DNA (and also proteins and lipid), damaging epithelial cells of the basal layer, and HPV-infected sperm cells, oocytes and embryos. Blastocysts transfected with DNA of HPV16 (only, not HPV18, or 31, or 33) demonstrate significant fragmentation of genomic DNA [100]. DNA damage causes an enhanced death by apoptosis of extravillous trophoblast cells transfected with HPV16 DNA (plasmid containing the entire HPV16 genome) [61] which may mimic the outcomes of the transfer of HR HPV genome from the infected sperm cell into the oocyte. Transfection of sperm cells with exogenous E6/E7 encoding DNA of HPV16 and 31 also causes increased DNA breakage characteristic to the apoptosis registered by the fixed sperm comet assay [49].

On top of the oxidative stress caused by the activity of HR HPV oncoproteins (illustrated on the example of epithelial cells), the latest studies found that HPV infection can directly inhibit the functionality of aquaporin AQP8 involved in the elimination of excessive ROS [101]. AQP8 is important for the normal function of human sperm, as it takes part in cell volume regulation and end stage of cytoplasm removal during sperm maturation [102]. Inhibition of AQP8 resulting in the increased levels of ROS subjects sperm cells to additional oxidative stress, resulting in additional DNA damage. Naturally, the sperm and oocyte have mechanisms and enzymes that repair DNA damage; however, these mechanisms may fail to repair all abnormalities. The failure to repair can also be due to HPV infection [97].

HR HPV associated DNA damage may serve as a gate opener for the integration of viral DNA into the host genome [95]. The phenomenon of genomic integration of HR HPVs is well known. Repeatedly found and described in detail are integrations of genomic fragments containing blocks of early genes, specifically encoding oncoproteins E6 and E7. Early studies have shown that in vivo fertilized mouse embryos in vitro cultured to the blastocyst stage show preferential uptake of DNA fragments from the E6-E7 conserved region of several HPV types, including HPV16 and 18 [103]. One of the isoforms of HPV16 oncoprotein E6* not only increased the levels of ROS causing oxidative DNA damage, but also increased the frequency of plasmid DNA integration into the host genome of cervical keratinocytes as assessed by the colony formation assays [104]. Integration may occur as a consequence of DNA fragmentation with subsequent reparation attempts, which in the presence of fragmented HR HPV DNA may involve fragments of the viral genome. Oxidative damage to the host DNA facilitating integration of HPV DNA into infected cells causes further DNA damage [105].

HR HPVs differ in their propensity to integrate. This was repeatedly noted in the precancerous lesions and cancers. For example, a genome-wide profiling of HR HPV integration of cervical smears done using HPV capture technology demonstrated predominant integration of HPV16 and HPV18, rare integration of HPV33, 51, 58 and 59, whereas HPV30, 35, 39, 44, 45, 53, 56, 59, 74 and 82 were found only in the episomal form [106]. This falls in lines with observations of differential roles of HR HPVs in the induction of infertility, predicting HPV16 and 18 to have the strongest adverse effect.

Thus, the infertility may in part result from the DNA damage by ROS (RNS) generated in the course of HR HPV infection, not (fully) repaired by the cellular machinery compromised by HR HPVs, and aggravated by integration of HR HPV DNA. DNA damage in the sperm cell and/or the oocyte would reduce the viability of gametes, may cause their apoptosis, and interfere with fertilization and normal development of the embryo (Fig. 2).

## Infertility and anti-HPV immune response

The direct involvement of HPV infection into the male and female infertility is documented. However, the lately done epidemiological studies found no associations between HPV infection and unexplained recurrent miscarriage (RM)/spontaneous abortions. A retrospective case-control study of the cervical HPV infection in 49 women with RM and 475 women without any miscarriage and with at least one pregnancy at term detected HPV DNA in cervical smears of 61.89% (294) control women, but in only 26.53% (13) of women with RM [107]. Other studies found that placenta of women who became pregnant spontaneously (19.6%) and women not treated with in vitro fertilization (18.1%) tended to be not less, but more frequently positive for HR HPVs than in women treated with IVF (12.7%, *P* = 0.077) [108]*.* Also prevalence of HPV in the spontaneously aborted products of conception was found to be lower than in the placentas from the term deliveries, although the difference was not significant (17.7 and 24.4%, respectively, in age-matched study subjects) [109]. Overall, the adverse effects of HR HPVs on the spontaneous/preterm abortions (before 20 weeks of gestation) were found to associate more with the absence of HPVs than with their presence. This brings up an additional mechanism of HPV-associated infertility, namely immune-mediated clearance of HPV-infected cells of the reproductive system and/or the embryo.

### Innate and adaptive immune response against HPV

In over 80% of cases, HPVs are cleared by the immune system of the patients within two to three years post infection. During the early stages of HPV infection, the innate immune system recruits innate immune cells such as dendritic (DC), Langerhans (LC), natural killer (NK) or natural killer T cells, to create a pro-inflammatory microenvironment and restrict viral infection [110]. Cells of the innate immune system stimulate the induction of the adaptive immune response against HPV; NK cells are also able to directly eliminate HPV infected cells [111]. Only a minority (10–20%) do not effectively clear the virus and remain HPV DNA positive with a persistent active viral replication.

The success of HPV vaccination proclaims the primary role in HPV clearance (or rather inhibition of HPV infection/re-infection) of antibody response against the virus. Antibodies play a key role in neutralizing the virus whilst it resides on the basement membrane. Seroconversion in the natural genital infection results in the detectable neutralizing antibody to the major capsid protein L1 in the sera. Although seroconversion occurs in only 50–70% infected, is slow and level of antibodies is low [112], even a low level of anti-L1 protects against HPV infection [113]. Anti-L1 antibodies can block binding to cellular receptor and to the basement membrane, the latter can neutralize virus at extremely low concentrations. The other common explanation is protection by transudated serum antibody in the cervical secretions. Indeed, HPV vaccines induce anti-L1 antibodies that protect against the infection, with moderate to strong correlation between vaccine-induced antibody levels in the serum and in cervicovaginal secretions, indicating exudation/transudation of virus-neutralizing antibodies through the mucosal epithelium [114, 115]. In HPV-vaccinated individuals, transudation provides vaccine-induced anti-HPV antibodies at the mucosal level in concentrations neutralizing the virus, important role of these local/mucosal antibodies in the protection from HPV infection has been anticipated (see [116] for review).

Protection can be mediated by immune cells, but rely on anti-HPV antibodies. One possibility is the antibody-dependent cellular phagocytosis (ADCP) as a way to clear both virus and virus-infected cells, and also to facilitate antigen presentation and provide inflammatory mediators for the adaptive immune response [117]. In support of this option, monoclonal anti-HPV antibodies were found to contribute to the protection from HPV infection, at least in a mouse model [118]. They were shown to cross vaginal epithelium at the sites of micro disruptions, and protect mice against HPV challenge. ADCP augmented this protection, as the protection was less efficient in case of passive transfer of F(ab′)2 instead of whole IgG in Fcγ-deficient mice; and in mice depleted of neutrophils and Gr1+ macrophages [118]. The other possibility is antibody-dependent cell-mediated cytotoxicity (ADCC). Friedman J et al. has shown that HPV-infected tumor cells can be directly killed by high affinity NK-cells, with killing potentiated by ADCC involving anti-PD-L1 antibodies [119]. A similar mechanism can be realized for the NK-cell killing of HPV-expressing cells via ADCC dependent on HPV-specific antibodies.

Not less important is the T cell response. Anti-HPV cytotoxic T-lymphocyte (CTL) response eliminates HPV-infected cells that had escaped neutralization by antibodies. In fact, T-cell response may represent the key mechanism of elimination of HPV infected cells. This is supported by previous observations on vaccinees who remained protected against HPV infection after waning of detectable antibody titers [120]. HPV epitopes targeted by T-cell response are well mapped: proteome resource website http://cvc.dfci.harvard.edu/hpv contains currently known T-cell epitopes of HPV with restriction to the ligands of human leukocyte antigens (HLAs). Analysis of these data shows that during acute HPV infection, the immune response is focused on L1 and L2 proteins, in the intermediate phase, on E4 and E5, and in the persistent infection, on HPV oncoproteins E6 and E7 [121, 122]. L1-specific proliferative CD4^+^ and CD8^+^ T-cell responses are also induced after HPV vaccination [123].

The “late” epitopes derived from the E6/E7 oncoproteins are recognized by both peripheral and tumor-infiltrating CD8+ T cells [124–126]. Improved survival of patients with HPV-related oropharyngeal cancer associates with tumor-infiltrating CD8+ T cells specific to E6/E7 [124, 127]. In cervical neoplasia and cancer, CD8+ T cell reactivity to E6 peptides appears to be dominant across all disease grades, inferring that E6-specific CD8+ T cells are not vitally involved in HPV clearance, whereas frequency of CD4+ responders is far lower among those with progressive disease, indicating the importance of CD4+ T-cell response for HPV clearance [87]. Indeed, multiple CD4+ T cell epitopes of HPV16 E2, E5, E6, E7 proteins restricted to nine HLA-DR restricted alleles were identified in healthy donors [128]. Such T-helper cell response does not necessarily has a lytic function, but may be critical for the generation and maintenance of the protective B-cell responses, as well as the induction of CTL response.

Lately, significant associations were detected between HLA class II alleles/haplotypes and outcomes regarding HPV clearance or persistence, with certain alleles/haplotypes favorably associated with viral clearance and prevention of HPV redetection (reinfection) [129]. Altogether, these data indicate that people having these alleles/haplotypes would be capable of efficient presentation of viral peptides activating potent immune response and facilitating clearance of HPV-infected cells.

### Infertility and antibody response against antigens exposed on the sperm cells

The role of anti-HPV immune response in infertility is basically uninvestigated. Single widely accepted immune mechanism of infertility is via anti-sperm antibodies (ASA). In one of the earliest studies, Menge AC et al. revealed ASA in 16.5% of men and 21.6% of women in 698 infertile couples [130]. Overall, in 31.1% of the couples at least one partner was positive for anti-ASA. Incidence of pregnancy was significantly reduced in infertile couples where both partners had anti-ASA in serum and/or genital tract secretions. Titers of ASA were significantly correlated to the reduced sperm penetration into the cervical mucus; sperm immobilizing activity was detected in 29.6% of the cervical mucus samples from 459 women [130]*.* The latest reviews report ASA in almost 9–12% of patients who are infertile due to different causes. ASA are not induced by the post-coital presence of spermatozoa in the reproductive tract of women, but may be caused by trauma to the vaginal mucosa, or by anal or oral sex resulting by processing of sperm antigens in the gut [131]. It is strongly believed that the infertility in humans and other species is related to a sub-population of anti-sperm antibodies which bind to sperm antigens; this concept laid grounds to the development of contraceptive vaccines based on ASA antigens [131].

The information on the effect on male and female fertility of anti-HPV antibodies is sparse. Of note, HPV-infected women were reported to have an increased level of ASA. It was then suggested that HPV infection may enhance the production of anti-sperm antibodies resulting in the elimination of HPV-infected sperm cells [132]*.* In this scenario, HPV-infected sperm cell acts as a carrier for HPV antigens exposed on its surface along with the sperm antigens. In HPV-free women naïve to HPV, HPV-loaded sperm cells would initiate, and in HPV-experienced, boost humoral immune response against HPV. Repeated vaginal expositions to such sperm would serve as repeated boosts, eventually resulting in the antibody mediated elimination of HPV-infected sperm cells. Acting as a carrier, HPV-loaded sperm cell may also break tolerance to the sperm antigens by epitope spreading (phenomenon of epitope spreading [133]). ASA and anti-HPV antibodies would then compromise the viability of not only HPV-infected, but also of un-infected sperm cells. The relation between ASA and anti-HPV antibodies, and their relative inputs into the infertility has yet to be determined.

### Possible immune-mediated mechanisms of HPV-associated infertility

Some of the cases of spontaneous abortions and lower prevalence of HPVs in women suffering from RM may be explained by the immune response against HPV, attempting to protect the host against HPV infection which ends up with affecting HPV-infected fetus. The experimental evidence on the direct involvement of anti-HPV immune response in infertility is currently missing. However, an indirect evidence has accumulated in the form of the associations of positivity for HPV DNA with the early loss of pregnancy, on contrary to the inverse correlations between positivity for HPV DNA and rates of late spontaneous abortions (miscarriage). In a retrospective case-control study, 49 women with unexplained recurrent miscarriage (RM) and 475 women without any miscarriage and with at least one pregnancy at term were checked for the cervical HPV infection [107]. Women with recurrent miscarriage had lower prevalence of HPV+ DNA tests than controls (26.53% (*n* = 13) of RM and 61.89% (*n* = 294) of the control women). Difference was highly statistically significant (*P* < 0.001) for the women in the 30–39 years age range. Women with and without RM did not differ in the detected HPV types, cytological or histological findings. This led Ticconi C et al. to the hypothesis that the recurrent miscarriage was caused by the immune response against HPV, protecting the host against HPV infecting the reproductive system and the fetus, success in protection reflected by low prevalence of HPV DNA [107].

Interestingly, prevalence of HPV, specifically of high oncogenic risk ones is the highest among young women, up-to 50%, but significantly decreases with increasing age, falling to 20% among 50- to 54-year-old women. Same tendency is observed for the HPV-associated cervical dysplasia/neoplasms [134]. Our study of women living with HIV-1 have also found positivity for HR HPV DNA to be the highest among women younger than 29 years, and the lowest among women aged > 39 (Nosik M, Isaguliants M, Palefsky JM, submitted). These observations could be explained by the gradual, with age, accumulation in women (men as well) of anti-HPV immune response, mediating HPV clearance and hindering re-infection (see [135] for a comprehensive review). These anti-HPV antibodies may keep mother protected from HPV infection, and at the same time interfere with the development of HPV-infected fetus, explaining low prevalence of HPV DNA in women with RM.

So far, the studies of immune-mediated mechanisms of HPV-associated infertility addressed only the humoral arm of anti-sperm immune response, but presence of the cellular arm, anti-HPV CTL and lytic T-helper cells, ADCC or ADCP cannot be excluded. FISH analysis of the round cell population in the semen confirmed presence of HPV DNA in CD45+ leukocytes. HPV DNA containing cells also displayed HPV16 E6 and HPV16 L1 viral proteins and, upon further investigation, were found to be CD20+ and CD56+, i.e. phenotypically, B and NK cells, respectively [136]. B lymphocytes are professional antigen-presenting cells despite their primary role in the humoral immunity [137]. Activated human NK cells express HLA-DR and can initiate MHCII-dependent CD4+ T cell proliferation (although competing with DCs) [138]. Placenta harbors broad population of macrophages, in villitis resident macrophages, Hofbauer cells, are intermixed with infiltrating maternal macrophages and CD8+ T cells with an inflammatory transcriptome resembling the biological processes that occur during antigen presentation and subsequent adaptive immune response [139]. The role of cellular immune response against HPV antigens expressed by the cells of the reproductive system in male and female infertility remains to be elucidated.

Thus, both male and female infertility may be associated with anti-HPV immune response mediating clearance of HPV-infected spermatozoa, oocytes, blastula and blastocytes at the early, and the immune rejection of the HPV-infected embryo as the maternal graft-versus-host disease against HPV infected fetus, at the late stages of embryogenesis (Fig. 2).

### HPV-associated infertility and HPV vaccines

HPV vaccination of girls/adolescent women protects them from acquisition of the most common HPV types, including HR HPVs (in Gardasil 4, in Gardasil 9, more in the latest vaccine variants). Vaccination would preclude acquisition of the virus from HPV-infected partner(s), with subsequent infection of placenta and oocytes. Studies in men demonstrate that they are equally vulnerable, as HPV infection seriously affect their fertility, both with regards to the viability and quality of sperm cells, and their capacity to fertilize women, specifically on the background of natural anti-HPV immune response. This strongly supports the necessity of broad HPV vaccination of both men and women, not only to protect them from HPV-associated cancer, but also to ensure their reproductive health.

This was excellently supported by an adjuvant HPV vaccination performed on 151 infertile couples with detection of HPV in semen [140]. Half accepted vaccination (vaccine group, *n* = 79) whilst the other did not (control group, *n* = 72). HPVs were detected in semen by INNO-LiPA and FISH 6 and 12 months post basal evaluation. Forty-one pregnancies, 11 in the control group and 30 in the vaccine group, were recorded (respectively 15% and 38,9%, *p* < 0,05) and resulted into 4 deliveries and 7 miscarriages (control group) and 29 deliveries and one miscarriage (vaccine group, *p* < 0,05 vs control group) [140]. Adjuvant vaccination associated with enhanced HPV healing of semen cells from HPV: in vaccinated group, percentage of HPV DNA+ semen samples reduced 8- and of HPV DNA+ exfoliated cells, 10-times, and level of anti-ASA antibodies, 2-times compared to the control group. The difference was significant for the vaccinees for whom treatment resulted in successful pregnancy compared to infertile (no pregnancy) controls [140]. HPV detection on sperms cells was predictive of negative pregnancy outcome, whereas adjuvant vaccination associated with increased rate of natural pregnancies and live births [140]. These results provide experimental proof of the negative effect of HPV infection on the reproductive health, and possibility to reverse it by HPV vaccination.

Thus, active immunotherapy of not only adolescent populations, but also of adults, can help to exterminate HPVs and restore reproductive health. Unfortunately, study by Garolla A et al. did not correlate healing of HPV and achievement of natural pregnancies to the dynamic changes in anti-HPV immune response. However, earlier HPV vaccinations in the adult population done by this group demonstrated that vaccine responses can be induced only in part of the HPV infected men, while others remain to be seronegative (are non-responders) [141]. After vaccination, subjects seropositive at recruitment showed absence of multiple infections and reduced prevalence of HPV semen infection in longitudinal post-vaccination survey (12 (*P* = 0.039), 18 (*P* = 0.034) and 24 months (P = 0.034), indicating boost of antibody response against HPVs [141]. Correlates of immune response or no response to HPV vaccination in adults previously exposed to HPVs are yet unknown.

CDC now recommends catch-up HPV vaccination for all persons up to the age of 26 years. For adults aged 27 through 45 years, public health benefit of HPV vaccination is minimal. A shared clinical decision-making is recommended as some persons who are not adequately vaccinated might benefit [142]. Obviously, infertile couples belong to the populations in need of such vaccination, although further studies are needed to understand how to (re)induce anti-HPV immune response in adult population, specifically in non-responders to standard vaccination protocols. The key message is that HPV vaccination/catch-up vaccinations were conclusively shown NOT TO AFFECT the fertility (evidence does not suggest any causal relationship between HPV vaccination and infertility; see WHO report https://www.who.int/publications/m/item/human-papilloma-virus-vaccines-and-infertility). Furthermore, it can prevent HPV-associated infertility, and even reverse it for those already HPV infected.

## Conclusions

The data reported in this review strongly support a multi-facetted role of HPV infection in all stages of human reproduction, delineating the important in-puts of oxidative stress induced by high risk HPV types, specifically HPV16, causing DNA damage and genomic instability, and of the immune response against HPV-infected cells of the reproductive system and HPV-infected embryo. The current high frequency of human reproductive impairment associated with HPV infection, and the availability of highly effective and safe prophylactic anti-HPV vaccines, strongly support the implementation of female and male vaccination not only to reduce HPV-associated oro-pharyngeal and ano-genital cancers, but also to reduce (or even eliminate) the HPV-associated human reproduction impairment and give a better opportunity to the human population to enjoy a healthy reproductive future.

## Data Availability

Not applicable.

## Abbreviations

ADCP: Antibody-dependent cellular phagocytosis
ADCC: Antibody-dependent cell-mediated cytotoxicity
ASA: Anti-sperm antibodies
CTL: Cytotoxic T-lymphocyte
DC: Dendritic cells
eNOS: Endothelial nitric oxide synthase
the EqS: Equatorial segment of mammalian sperm
GWAS: Genome-Wide Association
HBC: Hofbauer cells
HLAs: Human leukocyte antigens
8-OHDG: 8-Hydroxyguanosine
HR HPVs: High-oncogenic risk HPV types
iNOS: Inducible nitric oxide synthase
*ISH*: In Situ Hybridization
ICSI: Intra-cytoplasmic sperm injection
IUI: Intrauterine insemination
IVF: In-vitro fertilization
LC: Langerhans cells
MGT: Male genital tract
NK: Natural killer cells
NOS: nitric oxide synthase
nNOS: Neuronal nitric oxide synthase
NOX: Nicotinamide adenine dinucleotide phosphate oxidase
PCOS: Polycystic ovary syndrome
POF: Premature ovarian failure
ROS: Reactive oxygen species
RNS: Reactive nitrogen species
RM: Recurrent miscarriage
SSC: Spermatogonial stem cells
